# Probability of Low‐Altitude Midair Collision Between General Aviation and Unmanned Aircraft

**DOI:** 10.1111/risa.13368

**Published:** 2019-07-10

**Authors:** Anders la Cour‐Harbo, Henrik Schiøler

**Affiliations:** ^1^ Department of Electronic Systems Aalborg University Aalborg East Denmark

**Keywords:** Midair collision, modeling, safety, unmanned aircraft

## Abstract

Unmanned aircrafts (UA) usually fly below 500 ft to be segregated from manned aircraft. However, while general aviation (GA) usually do fly above 500 ft in areas where UA are allowed to operate, GA will at times also fly below 500 ft. Consequently, there is a distinct risk of near‐miss encounters as well as actual midair collisions (MACs). This work presents a model for determining this risk based on physical parameters of the aircraft and actual figures for the numbers of GA in a given airspace, as well as the probability of having GA below 500 ft. The aim is to achieve a prediction with a precision better than one order of magnitude relative to the true MAC rate value. The model is applied to Danish airspace and the MAC rate for unmitigated operations of UA is found to be approximately 10^−6^ MAC per flight hour. The model is particularly well suited for beyond visual line‐of‐sight operations, and is useful for UA operators for conducting risk assessment of planned operations as well as for regulators for determining appropriate operational requirements.

## 1 INTRODUCTION

The operations of light commercial unmanned aircraft (UA) are becoming more ubiquitous, as the various technologies necessary for operating longer, safer, and more accuratly are maturing. As the number of UA increases in the airspace, the probability of midair collisions (MACs) between UA and other airspace users also increases. This work addresses the need for quantifying the risk that UA operating beyond the visual range of the operator pose to airspace users that may occasionally or often operate at very low altitude. This is done by providing a model that predicts the probability of MAC for such scenarios. This model can be used by operators, manufacturers, and authorities for planning flight operations, for conducting risk assessment, for development of guidance material, and for aviation regulation. This work was in part prompted by the Danish Transport, Housing, and Construction Authority (DTCHA) in an effort to determine risk of MAC for beyond visual line‐of‐sight (BVLOS) operations in Danish airspace.

### 1.1 Background

As the number of UA in most national airspaces is growing rapidly, especially for amateur users, it has become evident through an increasing number of reports of near‐misses that collisions between UA and commercial aircraft are likely to occur, and the consequences could be catastrophic (FAA Center for Excellence for UAS Research, 2017a, 2017b). However, virtually all such cases are illegal operations where the UA have entered into controlled airspace, typically close to flight routes for commercial aviation. This type of flight operation is beyond the scope of this work.

But UA also pose a danger to general aviation (GA) operating in class G airspace below 500 ft where UA are indeed allowed to operate. As such, there is a distinct risk of MACs simply due to the fact that UA and GA (to some extent) share the airspace. Most GA operate solely according to visual flight rules (VFR) where the primary method for maintaining separation between airspace users is visual navigation. When operating BVLOS, UA are generally not able to comply with this method, and technologies that allow UA to reliably detect GA (such as radar, LIDAR, cameras) are still both quite expensive and somewhat immature. For this reason, it is valuable to estimate in a quantitative way the actual probability of MAC. This estimation will allow authorities, aircraft manufactures, and operators to assess the risk, and plan and execute flights accordingly.

### 1.2 Previous Work

The vast majority of previous work in MAC modeling is associated with manned aviation, in particular with scenarios where aircraft are in controlled airspace following directions from either ATC (air traffic control) or following predetermined flight patterns. This is reviewed in Section 1.2.1. The main aim for such models is how to maintain sufficient separation between aircraft without being overly conservative and thus waste precious airspace. The literature on GA MAC modeling is somewhat more limited, and tends to focus on risk associated with pilots not being sufficiently observant during flight. There is rather little literature related to modeling of UA colliding with GA. Some sources are reviewed in Section 1.2.2.

#### 1.2.1 Manned Aircraft Collision Modeling

Different models for determining the probability of aircraft actually colliding during flight have emerged over the years. Many date back to the 1960s and 1970s where the rise in air traffic over some parts of the globe made it necessary to thoroughly consider how to avoid MAC, and as part of this process, how to determine the likelihood of MAC. These models focus on aircraft following predefined air corridors, such as cross‐Atlantic flights. Some of the first to consider collision risk for such operations were Marks (1963) and Reich (1966), presenting a model for the probability of MAC for aircraft following a predetermined route. The Reich–Marks model was based on the assumption that there are random deviations of aircraft positions and speeds from those expected. Another early example is Machol (1975), which examines the collision risk over the North Atlantic for increasing commercial airline traffic when using designated flight rules for lateral and vertical separation. The work proposes a model specific for the risk associated with this type of flight. One of the most comprehensive models for MAC is Endoh (1982), which derives a first principle's model for collisions based on aircraft density, speed, and aircraft size. The result is a stochastic model for predicting MAC rate for airspace with known parameters for traffic. A model for vertical separation of manned commercial aircraft in U.S. airspace is presented in Richie (1989). It accounts for planned movements using flight plans and tracking data. The aim is to find the frequency of pairs of aircraft flying at above FL290 being near each other; near being defined as less than 1,000 ft vertical separation. A stochastic model is presented in Datta and Oliver (1991) for MAC between both commercial and GA as a function of the type of control airspace in which they are operating as well as aircraft velocities, geometries of approach patterns, and aircraft density. The work concludes that by far the most risky flight appears to be GA interacting with other GA in positively controlled environments. In Brooker (2003), a model is presented based on the Reich model, where some of the (claimed) shortcomings of that model are addressed. This is done by taking an event‐based approach, where the parameters directly reflect physical properties of the aircraft flight. In Blom, Bakker, Everdij, and van der Park (2003), human factors and technical factors are included in modeling, which is stochastic in nature. The purpose is to conduct an assessment that takes ATM into account rather than focusing narrowly on aircraft nonnominal behavior, as most other models do. MAC risk in high‐density airspace is investigated in Montanari, Baldoni, Morciano, Rizzuto, and Matarese (2012), which presents a model using cylindrical approximations of the aircraft. This is a purely geometrical model that works in three‐dimensions (3D) by means of projections. As such, this model can handle altitude changes of aircraft. The work also provides an example taken from airspace at Maastricht. In Fujita (2013), an expansion of the Rice model includes more detailed flight trajectories and time‐dependent position errors. It also includes aircraft kinematics and wind.

A description of the development of collision risk models for commercial aviation from the 1960s to 1995 is given in Machol (1995), and it details how they have been applied to safe separation standards for various geographical areas around the globe over a period of 30 years. The focus is on models for commercial air traffic. A recent review of MAC models for commercial aviation is Netjasov and Janic (2008), which includes descriptions of the general principle for many of the employed models. A very comprehensive review of 33 different ground risk models is given in Washington, Clothier, and Silva (2017). The review categorizes the models into failure, impact, recovery, stress, exposure, incident stress, and harm models, and compares them on a number of parameters.

Although there is plenty of literature on the risk and mitigation of MACs involving GA, there is less literature on the modeling of probabilities of MAC involving GA. The main focus in the GA‐related literature is on VFR and how that particular method of risk mitigation relates to collision avoidance. As an example, Koglbauer (2015) describes a training method for enhancing pilots' ability to avoid MAC during VFR flight, and Alexander (1970) looks at the probability of MAC under VFR and models the risk as a function of aircraft density in relation to land area.

#### 1.2.2 UA MAC

A data‐driven approach is presented in McFadyen and Martin (2016a, 2016b), where flight data from manned aviation are used to determine optimal exclusion zones for UA. This method focuses on low‐altitude high‐density airspace, and uses the data to generate polygonal zones dividing the airspace into density categories. Maki, Weinert, and Kochenderfer (2010) present a similar approach that uses recorded surveillance data and intended flight paths to estimate the probability of collisions. Both Weibel, Edwards, and Fernandes (2011) and Adaska (2012) present a model based on prediction of future “intruder” trajectories through a stochastic process. The former proposes a boundary in both time and distance for well clear separation in compliance with a rigorous safety assessment. The latter develops the algorithmic tools for computing the risks of the former by expanding techniques developed in the target tracking community. Melnyk, Schrage, Volovoi, and Jimenez (2014) propose a framework to develop effectiveness requirements for any sense‐and‐avoid (SAA) system by linking UAS characteristics and operating environments to MAC risk quantified by means of a fatality rate. The framework is based on a target level of safety approach using an event tree format. The framework of Jamoom, Joerger, Khanafseh, and Pervan (2015) is also based on SAA capabilities for an aircraft facing an intruder. This is done by defining integrity risk and continuity risk as UAS SAA safety performance metrics. The article provides methods to evaluate these risks along with requirements to ensure a predefined level of safety. The article also maps and bounds the trade space of requirements necessary to maintain desired integrity and continuity. Alfredson, Hagström, and Sundqvist (2015) present a study (a part of the European MIDCAS project) on operations of UA in controlled airspace, where the UA is equipped with detect and avoid functionality. The focus is on situation awareness, and the article reports the lessons learned from three human‐in‐the‐loop simulation campaigns. A stochastic Monte Carlo model is used in Patlovany (1997) to compare the relative MAC course probabilities and mean closing velocities. The article claims that regulations in the United States in 1997 can be shown to increase probability of MAC. This work is not for UA, but is applicable due to its generic nature. A simple model based on movements of particles is given in a thesis (Awad, 2013), which also provides some statistics on MAC between manned aircraft. The thesis presents first‐order models adapted from the literature that estimate the MAC risk, and model verification is achieved through extensive analysis of historic civil aviation accidents. The two conclusions are that (1) UAS already achieve manned levels of safety with respect to MACs because GA aircraft routinely operate in conditions where see‐and‐avoid is used but is not effective, and (2) the risk due to ground collision of UAS is sufficiently small to allow operations over the majority of the United States. Finally, two survey papers (Chand, Mahalakshmi, and Naidu, 2017; Mahjri, Dhraief, and Belghith, 2015) provide an overview of collision avoidance systems. The former gives a brief review of the components of SAA, while the latter is a somewhat more comprehensive tutorial that outlines and reviews the substantial breadth of SAA architectures, technologies, and algorithms.

### 1.3 Current Work

The focus in this work is to develop a model that can predict the probability of a MAC between a UA and any form of GA in class G airspace with relatively good accuracy. The model is contingent on the GA operating “randomly,” that is not following specific flight routes or patterns, or that such patterns are unknown at the time of modeling.

A quite thorough first principle approach similar to the one used in this work was derived in Endoh (1982), albeit using a slightly different method for derivation, as well as deriving more detailed variations of the model. That work did not address any particular composition of the airspace users, such as this work does. In addition, the model structure in this work is designed to accommodate information that (at least in theory) should be relatively simple to obtain, and it allows for varying degrees of specificity depending on the level of detail of the information about the UA and the GA.

Unlike most existing models, the method proposed in this work is particularly well suited for BVLOS operations where the UA operator is not able to visually segregate the UA from any GA, and where no other effective means of detect and avoid are in place. Also unlike most other works, we do use the model to compute MAC rates for a specific airspace (mainland Denmark) to both demonstrate the use of the model and to show the estimated MAC rates for various types of GA.

The aim of this work is to achieve an estimated MAC rate that can reliably be expected to be no more than one order of magnitude from the true MAC rate. This aim is partly based on the expected quality of the data and partly on the identified model errors that follow from the simplifying assumptions listed below. In addition, manned aviation safety typically operates with orders of magnitude classification of levels of safety and levels of hazard, lending credence to our aim.

## 2 MAC MODEL

The model presented in this work is developed for GA such as small fixed‐wing planes, such as the Cessna 172 and Piper Cub; for small rotorcraft, such as the Robinson 22; for gliders, ultralights, hang gliders, balloons, etc., that could occasionally operate below 500 ft, and thus appear in the airspace where light commercial UA almost always operate. The model is probabilistic and is set up to accommodate parameters that can reasonably be expected to be available from national aviation authorities, or other similar sources of data.

The model structure and parameters are presented first in Section 2.1, followed by a detailed mathematical derivation of the model components in Section 2.2. The expansion of the model to include several different types of GA is explained in Section 2.3. Finally, in Section 2.4 we discuss the potential modeling error that the model assumptions may generate.

### 2.1 Model Components

The model is probabilistic in nature and relies on a number of parameters, some deterministic and some probabilistic. It is solely for determining the probability of collision between a UA and a GA. It does not include collateral damage on the ground from falling debris and the like. The basic concept of the model is presented in Section 2.1.1, followed by a description of the structure of the model in Section 2.1.2. In Section 2.1.3, descriptions of all required parameters are given.

#### 2.1.1 Modeling Concept

The initial model setup is for a single UA and a single GA. Later, the model will be expanded to include multiple GA. The UA and the GA are represented by vertical cylinders approximating the physical extends of the two aircraft. The radius and height of the cylinders can either equal the width and height of the aircraft, or be safety zones for modeling near‐miss events. The model describes the probability that the “UA cylinder” will intersect the “GA cylinder.” Examples of cylinders are depicted in Fig. 1. The following additional assumptions also apply to the model.

1. The UA and the GA fly independently.
  **Figure 1:**
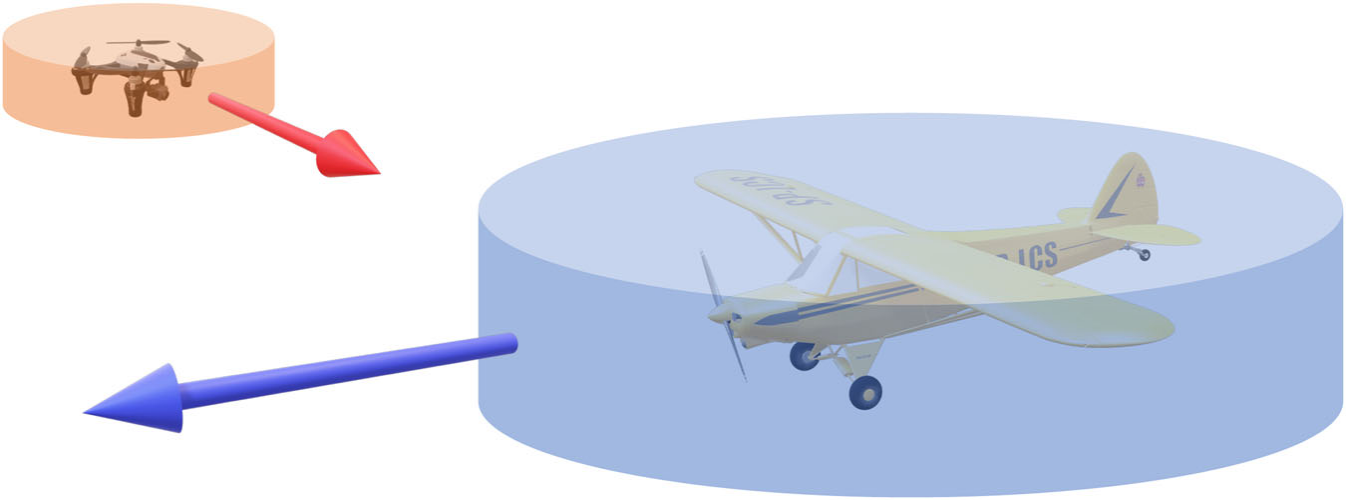
Two cylinders showing the modeled physical extend of the UA and the GA. The red cylinder is a UA flying in one horizontal direction, and the blue cylinder is a GA flying in another horizontal direction. A collision is defined as the two cylinders intersecting in space.
2. The UA fly below an upper altitude threshold.
3. The probability of the GA being below this upper altitude threshold is known (or estimated).
4. Flight directions for both aircraft are uniformly distributed from 0º to 360º.
5. Geographical positions of both aircraft are uniformly distributed across a predefined area.
6. Both aircraft fly only horizontally (no vertical movement) when close to each other.
7. Altitude of both aircraft is modeled probabilistically with predefined probability distributions.
8. GA is mostly not below 500 ft in any area where UA can legally operate. See Section 2.2.4 for details.

As a consequence of assumptions 4 and 5, the model is suited only for airspace where no external control is imposed to guide aircraft into certain patterns, typically class G airspace. Also, as a consequence of assumptions 6 and 7, collisions caused by sudden altitude change (i.e., altitude change during the time the two aircraft cylinders are overlapping horizontally) are not modeled. However, since the vertical speed typically is somewhat less than the horizontal speed, and since the vertically traversed distance for a GA is significantly smaller than the horizontally traversed distance, this assumption has only negligible effect for all types of GA but parachutes and balloons in descent or ascent. This is detailed in Section 2.4

The model supports fairly general assumptions on the type of GA (by averaging over typical size, speed, etc., for the entire population of GA), as well as more specific assumptions where data are available for individual types of GA. The latter is the approach taken in Section 3.

#### 2.1.2 Model Structure

The model for MAC is composed of four main probabilistic parts appearing in product form as:

(1) p MAC =p HC ·p VC ·p below ·λ STM .

The first term *p*_HC_ is the rate of “horizontal collision.” That is, the rate by which the two aircraft will be at the same two‐dimensional geographical location to allow a collision to occur. This rate is based on assigned circles around both aircraft (and a horizontal collision is defined as those two circles overlapping), and the assumption that both aircraft have known horizontal speeds.

The second term *p*_VC_ is the conditional probability of “vertical collision” given GA is below threshold. That is, the probability that the two aircraft are at the same vertical location (same altitude) and with zero vertical speed. This probability is based on conditional probability density functions for the altitude of both aircraft, as well as the height of both aircraft.

The third term *p*_below_ is the probability that the GA is below an altitude threshold, which in this work represents the maximum altitude at which UA would legally operate. How to determine an appropriate altitude is discussed later in Section 2.2.4.

The fourth and last term λ_STM_ is the effect of strategic and tactical mitigations. The three previous factors assume complete randomness in the aircraft motions (subject to the assumptions described above), and thus do not capture any mitigating means that could be introduced to reduce collision probability. However, for some flight scenarios there will be some form of strategic mitigations (i.e., mitigative means introduced before any flights and applying to all UA or GA) and/or tactical mitigation (i.e., mitigative means in effect during flight), and λ_STM_ captures the effect of such mitigations. Examples of strategic mitigation are temporal and spatial constraints, and examples of tactical mitigation are detect and avoid sensors and transponders as well as visual observations by the GA operator. In this work, λ_STM_ expresses how much the combined strategic and tactical mitigations will reduce the probability of collision. It is important to note that λ_STM_ as implemented in this model is only valid for relatively modest mitigations; it does not represent well relatively effective means such as operations in controlled or closed airspace.

These four products are largely independent; *p*_HC_ and *p*_VC_ are the probabilities of the GA and UA being at the same physical location in an North‐East‐Down coordinate system, where *p*_HC_ is the probability that the North–East coordinates sets for the GA and UA are equal (or sufficiently close for the cylinders to intersect) and *p*_VC_ is the probability that the Down coordinates are equal. Since the motions (and thus locations) of the UA and GA are independent, so too *p*_HC_ and *p*_HC_. The probability *p*_below_ is solely a property of the GA, and expresses the likelihood of the GA being in the same air volume as the UA, making it independent from *p*_HC_ and *p*_VC_. Finally, the last term λ_STM_ may have minor dependency on both *p*_HC_ and *p*_VC_ if, for instance, the flight altitude for the UA is chosen such as to mitigate the risk of colliding with known, say, glider, operations nearby. However, for most mitigations, such as transponders and SAA, there is rather little dependence on the altitude and/or geographical position of the GA.

#### 2.1.3 Model Parameters

Assume a given geographical area where the number and flight time of various GA are available, typically a nation. This area has size *G*. The number of GA available in this area is denoted as *n*. A GA has a given fraction of time airborne *T* (often provided as flight hours per year, which we denote as *T*_year_). It flies at an average speed *v*_GA_, and the GA cylinder has a vertical extend *h*_GA_ and horizontal radius of *r*_GA_. The UA similarly has values *v*_UA_, *h*_UA_, and *r*_GA_. The maximum altitude that we will consider is called *z*_max_. This can be 500 ft, but it can also be lower depending on the regional or national limit for UA operations. We assume SI units for all parameters.

### 2.2 Collision Model

The following derivation is for a single UA flying in an airspace with a single GA. It is composed of a horizontal component and a vertical component. The horizontal component is presented in Section 2.2.1, and the vertical component is presented in Section 2.2.3. Section 2.2.2 addresses how to exclude UA no‐fly zones such as airports. The altitude distributions of GA and UA are discussed in Sections 2.2.4 and 2.2.5, respectively. The risk mitigation factor λ_STM_ is briefly discussed in Section 2.2.6.

#### 2.2.1 Horizontal Collision Model

**Figure 2:**
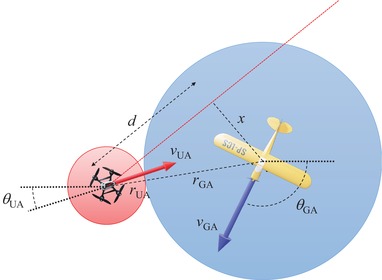
The figure shows the relative locations of aircraft cylinders as seen from above at the time of initial intersection. The thick arrows show the flight velocities of each aircraft. The red line shows the trace of the UA relative to the GA.

First, the average area covered by the cylinders (i.e., the disc resulting from the projection of the cylinders onto the horizontal plane) of one GA and one UA at any given time is:

G′=π(r GA 2+r UA 2)T,

and the fraction of the geographical area covered by the cylinders is $G^{\prime }/G$. The cylinders move over time as the GA and UA travel through the airspace, and the average time period the GA and UA cylinders intersect horizontally is:

(2) $$t_{i}=\frac{2\langle d\rangle }{\left\langle v\right\rangle },$$

where *d* is half the travel distance for the GA during safety zones intersection and *v* is the relative speed between the GA and the UA. See Fig. 2 for a visualization.

Now,

$$d\left(x\right)=\sqrt{r^{2}-x^{2}},$$

where r=r GA +r UA , and *x* is the distance between the center of the GA cylinder and the line that the center of the UA cylinder travels as perceived by the GA. We assume that *x* is uniformly distributed in the interval [0;r]. The average value of *d* then becomes:

$$\begin{array}{ccc} \left\langle d\right\rangle & = & \frac{1}{r}\int_{0}^{r}d\left(x\right)dx\\ & = & \frac{1}{r}{\left[\frac{1}{2}x\sqrt{r^{2}-x^{2}}+\frac{1}{2}r^{2}\arcsin\frac{x}{r}\right]}_{0}^{r}=\frac{r\pi }{4}. \end{array}$$

As *v* is the relative speed between the UA and GA, it is the norm of the difference of the two velocity vectors for the two aircraft:

(3) v=∥v UA −v GA ∥=v GA 2+v UA 2−2v GA v UA cos(θ UA −θ GA ),

where *v*_GA_ and *v*_UA_ are the norms, and θ_GA_ and θ_UA_ the angles (relative to a global frame) of ***v***_x_ and ***v***_GA_, respectively. Assuming that both θ_GA_ and θ_UA_ are uniformly distributed on [0;2π], the PDF for θ=θ UA −θ GA  is:

f(θ)=2π−|θ|4π2|θ|<2π0 otherwise .

The average value of cosθ in Equation (3) is then:

$$\left\langle \cos\theta \right\rangle =\int_{-2\pi }^{2\pi }\cos\theta f\left(\theta \right)d\theta =0,$$

and consequently,

⟨v⟩=v GA 2+v UA 2.

Inserting into Equation (2):

ti=(r GA +r UA )π2v GA 2+v UA 2.

Let $t_{o}$ be the average time the cylinders are not intersecting horizontally. Then the probability of intersection can be expressed as:

(4) $$p_{i}=\frac{G^{\prime }}{G}=\frac{t_{i}}{t_{i}+t_{o}},$$

and cylinder intersection rate is then:

(5) p HC (r GA ,r UA ,v GA ,v UA ,T)=1ti+to=piti=π(r GA 2+r UA 2)T(r GA +r UA )π2v GA 2+v UA 2G=2(r GA 2+r UA 2)Tv GA 2+v UA 2(r GA +r UA )G.

The unit for *p*_HC_ in this formulation is hertz. Note that this can be simplified by the assumptions r GA ≫r UA  and v GA ≫v UA , resulting in:

(6) p HC ≈2r GA Tv GA G.

We do not use this simplification in this work.

#### 2.2.2 Note on Area *G*

The geographical area *G* as defined above is typically a state or nation because states and nations have well‐known areas, and databases for GA typically cover specifically states or nations. However, it means that a certain fraction of *G* inevitably are areas where UA cannot legally operate (airports, security areas, etc.), and consequently Equation (5) will underestimate the probability of horizontal collision. Using in Equation (5) a smaller area $G^{*}\subset G$, representing the UA legally accessible area of *G*, would not remedy the problem as the yearly operational time *T* covers all flight of the GA, including flight outside $G^{*}$. However, under the (unsubstantiated, but arguably reasonable) assumption that the fraction $G^{*}/G$ approximately equals $T^{*}/T$, where $T^{*}$ is the flight time spent in $G^{*}$, Equation (5) still is a good approximation of horizontal impact probability, in particular because both $G^{*}/G$ and $T^{*}/T$ are close to 1.

#### 2.2.3 Vertical Collision Model

Under the assumption that the UA cylinder and the GA cylinder are at the same geographical location, we will now determine the probability that the cylinders intersect in the vertical direction. The vertical location (altitude) of both aircraft is at the vertical middle of the respective cylinders. Thus, there is a collision if the difference in altitude between the two aircraft is less than the sum of the cylinder heights.

The altitude of each aircraft is described by a probability density function, called f GA (z) and f UA (z) for the GA and UA, respectively. These are chosen so as to reflect how the aircraft actually behave in terms of altitude (see Sections 2.2.4 and 2.2.5). The probability that the GA and the UA are sufficiently close together in altitude for the cylinders to intersect is given by the joint distribution integrated over the joint space where the two altitude parameters are closer than half the sum of the aircraft heights. That is,

(7) p VC (f GA ,h GA ,h UA )=∫∫|α−β|≤h GA +h UA β<z max f GA (α)f UA (β)d(α,β)dβdα=∫0z max f UA (β)(F GA β+h GA +h UA 2−F GA β−h GA +h UA 2)dβ,

where *F*_GA_ is the cumulative distribution function associated with *f*_GA_. The basis for choosing a particular *f*_GA_ and *f*_UA_ is discussed in Sections 3.1.3 and 3.1.4.

The vertical collision model (Equation (7)) does not handle the limits very well, in the sense that the model implicitly uses the vertical centers of both cylinders as reference. Thus, in this model at very low altitude and at altitude close to *z*_max_ the aircraft extend outside [0;z max ], which is not captured by Equation (7). So, if the cylinder heights are relatively large compared to *z*_max_, then *p*_VC_ is underestimating the intersection probability. However, for normal cases where the height of the aircraft is small relative to *z*_max_, this is a negligible issue.

#### 2.2.4 Probability of GA Below *z*_max_

In most countries, some geographical areas are designated as non‐UA areas (airports, aerodromes, helipads, nature sensitive areas, military areas, areas around certain buildings and installations, etc.). By definition, the complement to the set of these areas is the areas where UA can indeed fly. The model implementation has the assumption (as stated in Section 2.1.1) that GA is mostly not below *z*_max_ in any area where UA can legally operate. This is derived from the assumption that GA is generally required to stay above 500 ft, with only (some of) the areas listed above as exceptions. The model is also based on the assumption that in most cases when GA is indeed below *z*_max_ in rural or urban areas, it is not a normal operational situation (with balloons being an exception). Knowing approximately how often such abnormal operations occur allows for an estimate of how often GA is indeed below *z*_max_. As described above, the model (1) therefore includes the factor *p*_below_ that expresses the likelihood of a GA being below *z*_max_ (which does not have to be 500 ft, but should not exceed 500 ft). Estimated values for Denmark are shown and discussed in Section 3.1.

In addition, for GA flying below *z*_max_ we assume some altitude distribution *f*_GA_ for the purpose of determining probability of a vertical collision, as described in Section 2.2.3. In most cases, a uniform distribution between 0 and *z*_max_ is appropriate; as an example, a glider conducting an offsite landing will typically be on final approach when below 500 ft, and thus descend constantly until landing. Note that the probability density function for this distribution only covers the range from 0 to *z*_max_. This is because we assume for the vertical collision model that the GA is indeed below *z*_max_.

#### 2.2.5 UA Altitude Distribution

The UA will also be assumed to follow an altitude distribution *f*_UA_, which will depend on the mission flown. For long endurance missions for monitoring or transport, a normal distribution around the expected flight altitude seems reasonable, while for, say, a photographic mission for real estate or a sport events, a uniform distribution between 10 m and 50 m may be appropriate. Any distribution used should integrate to 1 over the interval 0 to *z*_max_.

#### 2.2.6 Strategic and Tactical Mitigations

The model in Equation (1) includes the factor λ_STM_, which is simply multiplied with the unmitigated probability of a MAC. The purpose is to allow a reduction of the MAC probability in case there are mitigations in place. Such measures may differ for individual UA and GA. This work does not address this in any detail, and the factor is primarily included to allow for relatively simple mitigative measures.

### 2.3 Multi‐GA Model

The MAC rate modeled in Equation (1) is valid for one GA. To account for multiple GA, we simply multiply the probability *p*_HC_ by the number *n* of similar type GA. Here, we assume that none of the airborne GA will have overlapping cylinders. The multiplication is a good approximation of the joint probability for many GA since the cylinders are quite small relative to *G*.

To have a model that captures jointly all GA in the airspace (ranging from fixed wing to balloons), we want to determine the four right‐hand side factors in Equation (1) for each type of GA. The parameters *v*_GA_, *r*_GA_, *h*_GA_, and *f*_GA_ will be different for different types of GA, and must be inserted in Equations (5) and (7) for each type. In addition, the probability *p*_below_ and the distribution *f*_GA_ will vary with different GAs, and so will the mitigation factor λ_STM_. These parameters must be quantified (either through measurements, estimations, or educated guessing) before the model is practically useful. Our approach to this is described in Section 3.1.1.

A good approximation of the joint probability for a MAC is a weighted summation of *p*_MAC_ from the model (1) for each type of GA. This does omit the fact that should a MAC occur between the UA and one type of GA, the probability of the UA colliding with another GA becomes zero. However, the approximation is quite good due to the overall low value for each *p*_MAC_.

The final model becomes:

(8) p MAC ≈∑i=1# of  GA  types p HC (r GA ,i,r UA ,v GA ,i,v UA )·p VC (f GA ,i,h GA ,i,h UA )·ni·p below ,i·λ STM ,i

where the sum runs over the different types of GA included in the model. Note that Equation (8) is the MAC rate for one UA operating in an airspace together with all GAs included in the model.

### 2.4 Model Error Assessment

The quantitative framework developed above for collision rate assessment is model based, and a number of simplifying assumptions were made for mathematical tractability. This of course introduces model errors, which manifest themselves into quantitative errors in the final result *p*_MAC_. From an overall perspective, event rates and probabilities appearing in risk analysis will often be of small magnitude, whereas the accuracy by which they are given is logarithmic. That is, if results predict the correct order of magnitude, they will most likely be acceptable. This aligns with the aim of this work, as stated in Section 1.3. Therefore, additive errors should be orders of magnitude lower than the results themselves, whereas multiplicative errors should be logarithmically low.

One significant simplification is the disregard of vertical flight speeds in the expression for $t_{i}$ in Equation (2). To assess the model error, consider a simplistic 3D model, where collision cylinders are replaced by 3D ellipsoids to allow for much simpler definition of collision. That is, a collision ellipsoid is defined as:

(9) $$x^{2}+y^{2}+{\left(4z\right)}^{2}=r^{2},$$

where the factor 4 reflects a vehicle height four times less than width and length. The escape time $T_{i}$ from the ellipsoidal center of a vehicle with a speed vector $\left(V_{x},V_{y},V_{z}\right)$ is found by:

$${\left(V_{x}T_{i}\right)}^{2}+{\left(V_{y}T_{i}\right)}^{2}+{\left(4V_{z}T_{i}\right)}^{2}=r^{2},$$

which gives

$$T_{i}=\frac{r}{\sqrt{V_{x}^{2}+V_{y}^{2}+{\left(4V_{z}\right)}^{2}.}}$$

The difference in escape time compared to $V_{z}=0$ is given by:

$$\begin{array}{c} \frac{\frac{r}{\sqrt{V_{x}^{2}\;+\;V_{y}^{2}\;+\;{\left(4V_{z}\right)}^{2}}}}{\frac{r}{\sqrt{V_{x}^{2}\;+\;V_{y}^{2}}}}=\frac{V_{x}^{2}+V_{y}^{2}+16V_{z}^{2}}{V_{x}^{2}+V_{y}^{2}}=1+16\frac{V_{z}^{2}}{V_{x}^{2}+V_{y}^{2}}. \end{array}$$

So, assuming $V_{z}^{2}$ to be at least 100 times smaller than $V_{x}^{2}+V_{y}^{2}$, we find $V_{z}$ accounts for at most 16% of the result. In addition, assuming that $V_{z}$ is only nonnegligible for a limited part of a normal flight, say 10% of the time, this further reduces the average error by that same factor. We get from Equation (4) that

(10) $$t_{o}=t_{i}\frac{1-p_{i}}{p_{i}},$$

so that p HC (r GA ,r UA ,v GA ,v UA ,T) in Equation (5) is similarly affected at most 1.6% (i.e., 10% of 16%) by neglecting a vertical velocity that is 10 times smaller than horizontal velocity.

Another simplification is the abstraction of GA and UA hull geometries to vertical cylinders. Obviously, two cylinders may intersect without a collision occurring, even if the cylinders are of minimal volume to contain the GA or UA. Thus, the probability of collision given cylinder intersection *P*_CI_ is less than 1, which directly affects the final result as a multiplicative error. However, *P*_CI_ is still relatively close to 1, since most GA as well as UA will appear as a predominantly filled rectangle (i.e., the projection of the cylinder) in the flight direction. Thus, *P*_CI_ and therefore the result is skewed much less than an order of magnitude (the aim of this work; see Section 1.3). Note that this error is reliably overestimating the probability of collision.

## 3 RESULTS

The predictive capabilities of the model are demonstrated using two common UA and a generic aircraft. The target region is Denmark, as the author has access to detailed data for Danish airspace. Similar results can be generated for other airspace as long as sufficient airspace data are available.

### 3.1 Model Parameter Values

To apply the model, a series of parameters are required. In the following, first the aircraft parameters are given in Section 3.1.1, and parameters for mitigation are given in Section 3.1.2. The distributions used for GA and UA are given in Sections 3.1.3 and 3.1.4, respectively.

#### 3.1.1 Aircraft Parameters

We will use nine different types of GA listed in Table I.

**Table I:** Properties of GA at Low Altitudes in Danish Airspace

	Number	Airborne	Speed	Radius	Height	Distribution		Probability <100 m	
Type	*n*	*T*_year_	*v*_GA_	*r*_GA_	*h*_GA_	*f*_GA_	Conditions for <100 m	*p*_below_	Example
Fixed wing <5,700 kg	700	100	75	6	2	U(0,100)	Emergency landing, special permits, illegal low flights, landing on temporary airstrip	0.1%	Cessna 182
Rotorcraft	115	100	56	5	3	N(100, 50)_[0; 100]_	Emergency, special permit, illegal low flight, temporary airstrip, tours	5%	Robinson 44
Glider	300	60	50	10	1.2	U(0,100)	Landing outside airport	1%	DG‐1000
Motor glider	135	175	50	9	2	U(0,100)	Emergency, landing outside airport	0.5%	ASH 26E
Ultralight	300	50	28	5	2.5	N(100, 50)_[0; 100]_		0.1%	Pegasus Quantum
Paraglider	320	20	15	6	6	N(50, 50)_[0; 100]_		0.5%	Gin Balero 6
Hang glider	80	20	20	5	1	N(50, 50)_[0; 100]_		0.5%	La Mouette Atlas
Parachute	800	5	8	3	7	U(0,100)	Occasionally outside airport	0.1%	SPEKON RL‐16/3
Balloon	75	20	10	30	64	N(100, 50)_[0; 100]_	Start, landing, enroute	10%	Cameron Z‐105

These types are provided by the DTCHA from its database of GA registered in Denmark, and the values for *n* are extracted from the same database. The parameters *v*_GA_, *r*_GA_, and *h*_GA_ are taken from typical GA in each category. The values *T* and *p*_below_ are estimates provided by Klavs Andersen and Anders Madsen from DTCHA. They are both aviation inspectors for UA and have extensive past experience from manned GA. There has been no additional review process or verification of the validity of their estimates. The authors have attempted to obtain independent figures for these two parameters from two other European national civil aviation authorities, but have faced significant reluctance to provide any estimates of this kind. This may be understandable given the ramifications erroneous estimates may have for aviation safety. The authors have also attempted to find historical statistics for these parameters, but these quantities do not seem to be recorded (in an accessible form) in available flight incident statistics. As described in Section 2.2.4, the probability values for *p*_below_ in Table I are applicable only outside restricted areas, such as airports, aerodromes, and helipads.

Since the maximum altitude in Denmark for flights outside urban areas is 100 m, we set z max =100.

Three different types of UA are used; a generic smaller fixed‐wing aircraft, the DJI Matrice 600 multirotor, and the larger fixed‐wing UAV Factory Penguin C. The parameters for the aircraft are shown in Table II.

**Table II:** Parameters for Three UA

Parameter	Generic	M600	Penguin C
*v*_UA_	18 m/s	6 m/s	21 m/s
*r*_UA_	0.8 m	0.5 m	1.7 m
*h*_UA_	0.3 m	0.6 m	0.8 m
*f*_UA_	U(0, 100)	N(25, 20)	N(90, 5)
Mitigation	n/a	Time restriction	DAA

#### 3.1.2 Mitigation

The values from mitigation are shown in Table III. There is no mitigation for the generic aircraft. The M600 flight uses time restrictions to avoid operating when conditions are right for balloons (during morning and afternoon hours), as well as knowledge that there is no paraglider, hang glider, or parachutes activity in the area. The Penguin C uses a radar‐based detect and avoid system that enables it to detect and avoid aircraft with a reasonable effectiveness. A mitigation factor of 1 will mean that no mitigation is in place. Do note that all the used <1 mitigative values are at present pure speculation and only serve to demonstrate the capabilities of the presented model.

**Table III:** Estimated Strategic/Tactical Mitigations for Three UA

	Strategic/Tactical Mitigation		
	Generic	M600	Penguin
Fixed wing <5,700 kg	1	1	0.5
Rotorcraft	1	1	0.5
Glider	1	1	0.5
Motor glider	1	1	0.5
Ultralight	1	0.2	1
Paraglider	1	0.2	1
Hang glider	1	0.2	1
Parachute	1	0.2	1
Balloon	1	0.1	0.2

#### 3.1.3 GA Altitude Distributions

**Figure 3:**
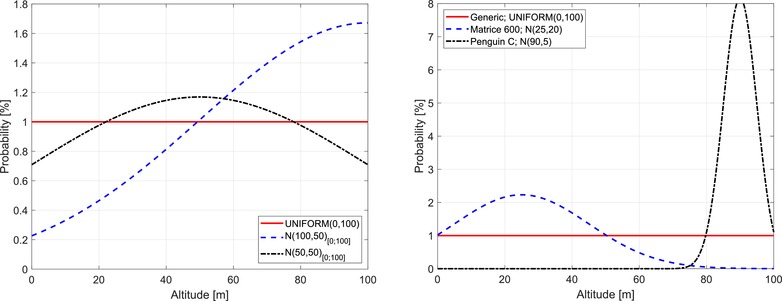
Left: The three PDFs used for the GAs listed in Table I. Right: The three PDFs used for the three UA scenarios. All are restricted to 0–100 m and capped as described in the text.

The GA distributions show how each GA type is distributed in altitude when below 100 m. This information is quite difficult to determine accurately, and in this work is based purely on considerations on how each type of GA could be expected to behave below 100 m. Three different distributions are used, a uniform between 0 m and 100 m, and two normal distributions, one with mean 100 m and one with mean 50 m. The used GA distributions are shown in Fig. 3. Both normal distributions are in fact cropped to the interval 0–100 m and scale to integrate to 1 over this interval.

Each GA type is assigned one of these distributions, as shown in Table I. Fixed wing, glider, and motor glide will typically be conducting a landing when at this low altitude, and their descent is assumed to be fairly constant. Therefore, a uniform altitude distribution is assigned. Parachutes also descent with a fairly constant rate, and are thus also assigned a uniform distribution.

Rotorcraft can occasionally conduct operations at lower altitude, but tend to stay some distance over the ground. The same goes for balloons, which tend to be some distance from the ground when below 100 m. Ultralights are powered and would typically also not fly low during most of the flight. All these GA are therefore assigned a normal distribution that puts them predominantly at the higher altitudes.

Paragliders and hang gliders are unpowered and are expected to operate around 50 m during the ascent and landing. As such, they are assigned a normal distribution around 50 m.

Do note that these distributions are speculative insofar as no research, to the best knowledge of the authors, has been done to actually determine such altitude distributions.

#### 3.1.4 UA Altitude Distributions

The altitude profile for operations of UA is obviously varying with the type of mission, and can be anything from flying at very low altitudes for action photography over changing altitudes for inspection to long endurance operations at maximum altitude for surveying. We will use three different profiles here:

1. Uniform distribution from 0 to maximum altitude, exemplified with a generic aircraft conducting flight operations at varying altitudes, and modeled with uniform distribution U(0,z max =100).
2. Low flight operations where the probability is decreasing with altitude, exemplified with a Matrice 600 conducting last‐mile package delivery, and modeled with N(25, 20), that is, a normal distribution with mean 25 m and standard deviation 20 m.
3. Near‐maximum altitude long endurance flight mission, exemplified with a Penguin C aircraft doing hub‐to‐hub parcel transport, and modeled with N(90, 5).

Both normal distributions are cropped to [0;z max ] and scaled to integrated to 1. The three distributions are shown on the right in Fig. 3.

### 3.2 MAC Rates

Using the parameters for Danish airspace shown in Table I and the parameters for the three different UA in Tables II and III, we can now compute the MAC rate both for the individual GA types and jointly.

**Figure 4:**
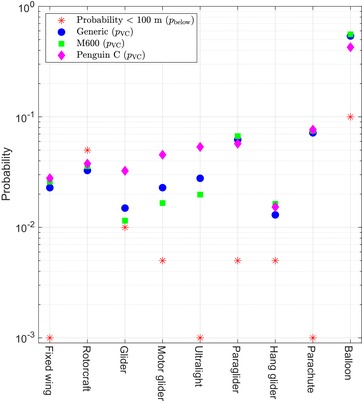
The probability of each type of aircraft being below *z*_max_ is shown in red (taken directly from Table I). The blue, green, and magenta dots show the probability of vertical collision *p*_VC_ as given by Equation (7) using the parameters given in Table I.

First, Fig. 4 shows the vertical collision model output *p*_VC_ along with the probability of each type being below *z*_max_. The *p*_VC_ numbers are shown in a separate plot since they are relatively high compared to *p*_HC_, and since they are probabilities (as opposed to rates).

**Figure 5:**
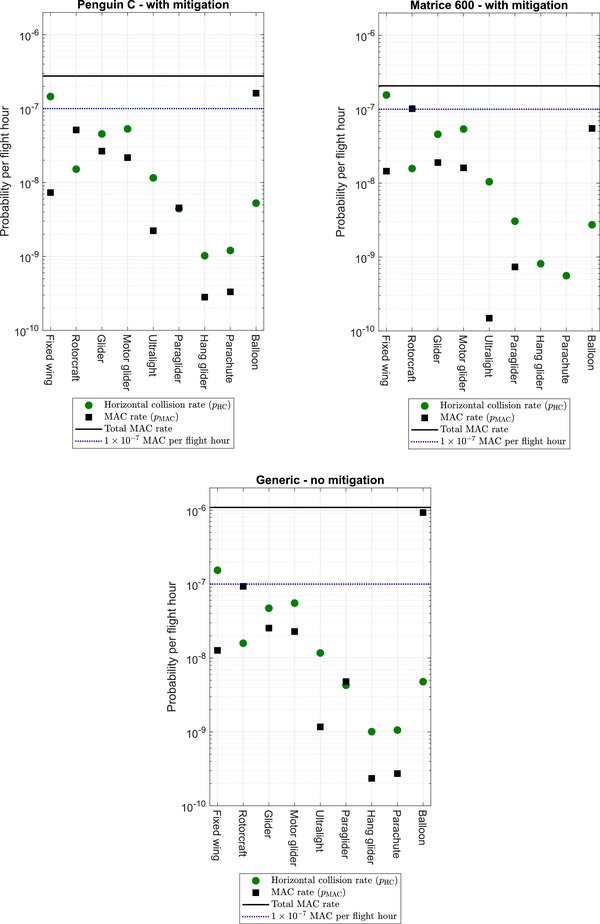
The green dots show the rates derived from the horizontal collision probability model (5). The black squares show the output of the MAC model in Equation (1) when multiplied with *n*, the number of each aircraft as listed in Table I, thus being the MAC rate. The solid black horizontal line shows the sum of the nine aircraft as given by Equation (8). For reference, the blue dashed line shows 10^−7^ MAC per flight hour.

Fig. 5 has a plot for each of the UA. Each plot shows the computed horizontal collision rates, along with the MAC rate, for all nine types of GA. The MAC rate is the output from the model in Equation (1). In addition, each of the three plots also show the approximated joint probability for MAC as computed in Equation (8), with $\rho_{i}$ set to 1 and $\rho_{i}$ set according to Table III, respectively. For comparison, the value 10^−7^ MAC per flight hour is also shown in each plot, as this is the order of magnitude that several sources report as being equivalent to the level of safety for GA (Dalamagkidis, Valavanis, and Piegl, 2008; FAA, 2013; Melnyk et al., 2014).

## 4 CONCLUSION

We have presented a model for predicting the MAC rates of UA operating at low altitudes in uncontrolled airspace. The model is targeted at MAC between one UA and all types of GA, and is designed specifically for altitudes below 500 ft. The model is conditioned on at least some knowledge of the GA airspace users (such as speed, size, and number), which may not necessarily be easy to obtain for a given airspace. However, this approach does provide some degree of certainty in the resulting probabilities in comparison to models based on stochastic assumptions only. The aim has been to achieve a precision of at least one order of magnitude relative to the true MAC rate.

The model is applied to Danish airspace and gives a generic MAC rate of approximately 10^−6^ collision per flight hour (black dashed line in “Generic” plot in Fig. 5). This relatively high value may come as a surprise, in particular that it is mainly a result of potential collisions with balloons. This also demonstrates that the total MAC rate is somewhat sensitive to the parameters chosen for the balloons (mainly the probability of occurrence below *z*_max_, and the strategic mitigations for avoiding balloons).

At the same time, the resulting MAC rates for the Matrice 600 and Penguin C scenarios are encouraging in the sense that they have the same order of magnitude as GA in itself, and thus comply with the concept of equivalent level of safety. In addition, they also largely comply with various recommendations in the literature, here specifically compared to 10^−7^ MAC per flight hour given by FAA (2013).

### 4.1 Reflections on the Model

The primary strengths of the model are (1) the ability to provide a reasonable estimate of MAC for any airspace where only standard data held by the aviation authorities are available, and (2) the applicability to the most common type of airspace that UA operate in, now as well as in the foreseeable future.

Although the model in Equation (1) is widely accepted and found in various forms in much of the existing literature, the derivation of the four individual terms may be subject to debate. We have divided the probability of an impact into horizontal and vertical “impacts,” which makes sense because the models for these are rather different and because aircraft generally behave differently horizontally and vertically. However, both GA and UA obviously move in 3D space and there is indeed a varying degree of correlation between the forward, sideways, and up/down motion for various types of GA and UA. This is not captured by the presented model. Also, the model does not capture collision caused by vertical motion, which is a distinct drawback, although we argue that for all but parachutes and balloons in ascent/descent, the modeling error is acceptable.

The assumption that GA is uniformly distributed in the relevant air volume is also a weakness of the model, insofar as UA pilots are aware of this and adapt their behavior accordingly (such as not operating at locations where aircraft are regularly observed, even though it would be legal to do so). However, while a model that would include such geographically specific information would not be that complicated to implement, it would probably be rather difficult to obtain reliable data for such a model.

### 4.2 Future Work

The primary effort in future work would be to address the separation of horizontal and vertical motions, that is, eliminating assumption 6 in Section 2.1.1. This would primarily be a matter of expanding the math to handle various types of cylinder intersection. It would be good for the precision to have better data to support *p*_below_ as well as λ_STM_, which at this point are estimations only.
